# Effectiveness of antibiotic treatment in children with Lyme neuroborreliosis - a retrospective study

**DOI:** 10.1186/s12887-022-03335-w

**Published:** 2022-06-09

**Authors:** Sigurdur Arnason, Barbro H. Skogman

**Affiliations:** 1 Department of Clinical Science, Intervention and Technology – CLINTEC, Alfred Nobels Allé 8, S-141 52 Huddinge, Sweden; 2 Department of Pediatric Infectious Diseases, Astrid Lindgren’s Children’s Hospital, Karolinska Vägen 22, S-171 64 Solna, Stockholm, Sweden; 3 Center for Clinical Research Dalarna – Uppsala University, Nissers Väg 3, S-791 82 Falun, Sweden; 4 Department of Clinical Science, Intervention and Technology – CLINTEC, Karolinska Institutet, Alfred Nobels Allé 8, S-141 52 Huddinge, Sweden; 5 Department of Medical Sciences, Örebro University, Södra Grev Rosengatan 42 B, S-703 62 Örebro, Sweden

## Abstract

**Background:**

Lyme neuroborreliosis (LNB) is a tick-borne infection caused by the spirochete *Borrelia burgdorferi* sensu lato complex with various neurological manifestations. The recommended treatment for LNB in Swedish children has been intravenous ceftriaxone 50–100 mg/kg × 1 (< 8 years of age) or oral doxycycline 4 mg/kg × 1 (≥ 8 years of age) for 10–14 days. Studies on adult LNB patients have shown equal efficacy for ceftriaxone and doxycycline, but no such studies have been conducted on pediatric LNB patients. The aim of this study is to retrospectively evaluate clinical outcome in children with LNB who have received intravenous ceftriaxone or oral doxycycline.

**Results:**

Clinical and laboratory data from three previously conducted prospective studies on children with LNB (1998–2014) were retrospectively analyzed. A total of 321 children (1–19 years of age), who received antibiotic treatment for definite LNB or possible LNB, were included. Clinical outcome at the 2-month follow-up (recovery/non-recovery) was evaluated using Chi^2^ test and logistic multivariate regression analysis. Out of 321 LNB patients, 194 children (60%) had received ceftriaxone and 127 children (40%) had received doxycycline. When comparing clinical outcome between treatment groups, no difference was found (*p* = 0,217). Results did not change when incorporating relevant clinical and laboratory data into the logistic multivariate regression analysis.

**Conclusion:**

In this large retrospective study, no difference in clinical outcome was found, independent of age, when comparing children who received ceftriaxone with those who received doxycycline, supporting an equal effectiveness for treatment of LNB pediatric patients. However, future randomized comparative treatment studies are warranted for evaluation of efficacy of antibiotic treatment in pediatric LNB patients.

## Background

Lyme borreliosis (LB) is the most common tick-borne infection in the northern hemisphere. The infection is caused by the spirochaete *Borrelia (B.) burgdorferi* sensu lato complex [1, 2]. The complex consists of at least 21 different species where the major human pathogens in Europe are *B. afzeli*, *B. garini and B. burgdoferi* sensu stricto [1–4]. When the spirochetes emerge from the skin into the central nervous system (CNS), symptoms of subacute meningitis and/or cranial or peripheral nerve impairment may occur, resulting in Lyme neuroborreliosis (LNB) [5]. The most common manifestation of LNB in children is facial nerve palsy followed by symptoms of subacute meningitis (fever, headache, neck pain, neck stiffness) [6–8]. Unspecific symptoms such as loss of appetite, change of mood or fatigue may sometimes be present in younger children [9]. Clinical outcome after treated LNB in pediatric patients is generally favourable, but persistent symptoms may occur and affect daily life in 13–20% of patients [10]. The results of a lumbar puncture, in addition to clinical symptoms attributable to LNB, are required to determine the diagnosis of LNB [11]. In the cerebrospinal fluid (CSF), pleocytosis (> 5 × 10^6^/L white cells with a mononuclear cell dominance) and intrathecal production of specific anti-*Borrelia*-antibodies are needed to confirm the LNB diagnosis [11].

In Sweden, the recommended treatment for LNB has been intravenous ceftriaxone 50–100 mg/kg × 1 (children < 8 years of age) or doxycycline p.o. 4 mg/kg × 1 (children ≥8 years of age) for 10–14 days, according to national guidelines. In latter years, the safety of tetracyclines has been in focus, mainly due to concerns of adverse effects such as dental staining and enamel hypoplasia in younger children. However, doxycycline has a lower calcium-binding capacity than previous generations of tetracyclines, and previous studies have shown that doxycycline is safe for children younger than 8 years of age [12–14]. Furthermore, oral doxycycline, contrary to intravenous ceftriaxone, is inexpensive, easy for parents to administer and hospitalized care is not needed [15]. Extensive use of cephalosporins causes negative effects on the bacterial flora (i.e. antibiotic resistence) and it is of outmost importance to limit the overall use of cephalosporins in the healthcare system [16].

In Norway, a non-inferiority trial was performed in 2007 on adult LNB patients. Participants (*n* = 118) were randomly allocated to receive intravenous ceftriaxone 2 g × 1 or oral doxycycline 200 mg × 1 for 14 days, 102 patients completed the study [17]. After 4 months, patients were evaluated for clinical outcome by using a composite clinical score (range 0–64, 0 = complete recovery and 64 = no recovery). In the doxycycline group, 26 out of 54 (48%) patients were completely recovered and in the ceftriaxone group, 16 out of 48 (33%) patients were completely recovered [17]. The study showed that oral doxycycline had an efficacy equal to intravenous ceftriaxone for treatment of LNB in adult patients [17]. Recently, another Finnish study by Kortela et al. was published, comparing oral doxycycline and intravenous ceftriaxone in adult LNB patients, further supporting these results [18].

In 2016 a systematic review was conducted on efficacy and safety of pharmacological treatments for LNB in children [19]. Two randomized-control trials and four non-randomized studies on treatment of LNB in patients younger than 18 years of age were included [19]. The authors conclude that there were no differences between the two strategies of antibiotic treatment (oral doxycycline versus intravenous beta-lactam antibiotics) in children with LNB, but the quality of evidence (GRADE) of the included studies was deemed very low. In addition, no evidence was found to support prolonged antibiotic treatment [19]. In summary, there is still an important gap in knowledge concerning the most efficient strategy for antibiotic treatment in pediatric LNB patients.

The aim of this study is to retrospectively evaluate clinical outcome in children who have received intravenous ceftriaxone as compared to children who have receive oral doxycycline as antibiotic treatment for LNB.

## Material and methods

### Design

Clinical and laboratory data from three previously performed prospective studies [7, 20, 21] were analyzed together in this retrospective study. The study periods were 1998–2001 [20], 2000–2005 [7] and 2010–2014 [21], respectively. Data from these three studies will be referred to as Cohort 1, 2 and 3 (Table 1). The three cohorts together represent a large portion of pediatric LNB patients at seven pediatric departments in a Lyme endemic area in central and southeast Sweden (Falun, Linköping, Norrköping, Jönköping, Västerås, Skövde, Lidköping), all of whom have received antibiotic treatment for LNB according to national guidelines. In each of the three previous studies, data were collected prospectively from structured questionnaires (in addition to information from medical records), on admission and at follow-up, and compiled in a database. In a few cases, a study nurse interviewed parents, from the structured questionnaire, over the phone, at the 2-months follow-up. Only patients with sufficient clinical data on LNB diagnosis and antibiotic treatment were included in this retrospective analysis. This explains why numbers of patients in each of the three cohorts are not congruent with numbers of patients in previous studies [7, 20, 21]. No patient was part of more than one cohort.

**Table 1:** Age, gender, known tick bite, erythema migrans and LNB diagnosis in the three cohorts of children with Lyme neuroborreliosis

	Cohort 1^a^	Cohort 2^b^	Cohort 3^c^
	(*n* = 77)	(*n* = 102)	(*n* = 142)
**Age, median (range)**	6 (1–18)	7 (2–15)	10 (2–19)
**Gender**			
Female, n (%)	30 (39)	45 (44)	71 (50)
Male, n (%)	47 (61)	57 (56)	71 (50)
**Known tick bite, n (%)**	42 (55)	59 (58)	82 (58)
**Erythema migrans, n (%)**	19 (25)	37 (36)	23 (16)
**LNB diagnosis**			
Definite LNB, n (%)	47 (61)	68 (67)	113 (80)
Possible LNB, n (%)	30 (39)	34 (33)	29 (20)

^a^ [20]

^b^ [7]

^c^ [21]

*LNB* Lyme neuroborreliosis

### Patient sample and follow-up questionnaire

The patient sample contained information about age, gender, symptoms, duration of neurological symptoms, diagnosis (definite LNB or possible LNB), antibiotic treatment (intravenous ceftriaxone or oral doxycycline; dose and duration of treatment), pleocytosis in CSF, intrathecally produced specific anti-*Borrelia* antibodies, and clinical outcome at 2-month follow-up (recovery/non-recovery). A total of 321 children between 1 and 19 years of age were included in the study. Patients were classified as definite LNB (*n* = 229) or possible LNB (*n* = 92). The diagnosis of definite LNB was defined according to the European case definition, by clinical symptoms attributable to LNB without other obvious reasons, mononuclear pleocytosis in CSF and intrathecally produced specific anti-*Borrelia* antibodies [11]. The diagnosis of possible LNB was defined by clinical symptoms attributable to LNB without other obvious reasons, mononuclear pleocytosis in CSF, absence of intrathecally produced specific anti-*Borrelia* antibodies, response to antibiotic treatment and no signs or laboratory findings of other disease. All definite LNB and possible LNB patients received and were clinically improved on antibiotic treatment and are considered as LNB patients.

At the 2-month follow-up, a structured questionnaire was used to document self/parent-reported persistent symptoms and a clinical examination was performed, including the House-Brackmann facial nerve grading scale (a physician-assessed six-point scale to evaluate facial nerve impairment) [22]. Based on this information, an evaluation was made by the pediatrician, as part of the analysis in each of the previous studies, and LNB patients were defined as being recovered/not recovered.

### Statistics

Statistical analyses were performed in IBM SPSS Statistics, version 26 (IBM Corporation, USA). When comparing continuous, non-normally distributed data between groups, the Mann-Whitney U test was used. For non-continuous data the Chi^2^ test analysis was used. A logistic multivariate regression analysis (generalized linear model), was used for all relevant clinical and laboratory variables with the dependent variable clinical outcome at the 2-month follow-up (recovery/non-recovery). A *p*-value of < 0.05 was considered significant.

### Ethics

The three previous prospective studies were approved by the Regional Ethics Committee in Linköping (Dnr 98,103 and Dnr 02–159) and Uppsala (Dnr 2010/106), Sweden. Every child was assigned a specific study-ID. Written informed consent was received from parents/guardians.

## Results

**Fig. 1:**
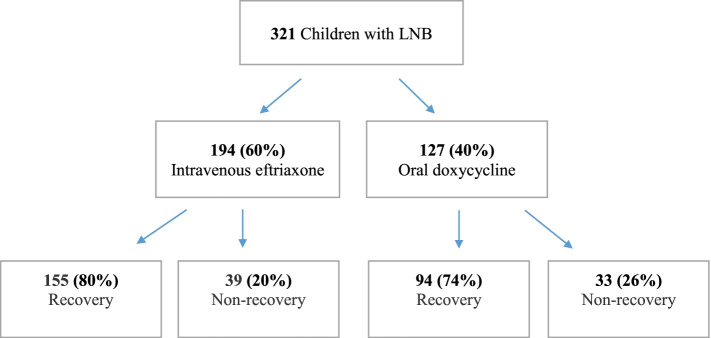
Flow chart showing different antibiotic treatment groups and clinical outcome as recovery/non-recovery in children with Lyme neuroboreliosis (LNB) at the 2-month follow-up

Out of 321 pediatric LNB patients, 194 children (60%) received intravenous ceftriaxone and 127 children (40%) received oral doxycycline (Fig. 1, Table 2). However, four patients had received both intravenous ceftriaxone and oral doxycycline during the treatment for LNB. Three of these patients were included as part of the ceftriaxone group, since the majority of their treatment was given intravenously. The fourth patient had received only one day of intravenous ceftriaxone followed by 13 days of oral doxycycline and was consequently included in the doxycycline group. In 38 LNB cases, the pediatrician had not followed the age recommendation and had chosen intravenous ceftriaxone instead of oral doxycycline for LNB treatment, even though the child was older than 8 years of age. Furthermore, one girl had received oral doxycycline, even though she was 4 years of age. No information about adverse events during antibiotic treatment was available.

**Table 2:** Age, gender and clinical characteristics of children with Lyme neuroborreliosis, on admission and at the 2-month follow-up in the two antibiotic treatment groups

	Ceftriaxone i.v.	Doxycycline p.o.
	(*n* = 194)	(*n* = 127)
**Age, median (range)**	6 (1–18)	10 (4–19)
**Gender**		
Female, n (%)	91 (47)	55 (43)
Male, n (%)	103 (53)	72 (57)
**Major symptoms and signs on admission**^a^		
Facial nerve palsy, n (%)	122 (63)	89 (70)
Headache, n (%)	121 (62)	89 (70)
Fatigue, n (%)	139 (72)	84 (66)
Loss of appetite, n (%)	93 (48)	58 (46)
Fever, n (%)	83 (43)	55 (43)
Neck pain, n (%)	70 (36)	54 (43)
Neck stiffness, n (%)	43 (22)	42 (33)
Nausea, n (%)	40 (21)	35 (28)
Radiant pain, n (%)	32 (16)	21 (17)
Vertigo, n (%)	19 (10)	21 (17)
**Duration of neurological symptoms on admission**		
1 week, n (%)	91 (47)	51 (40)
2 weeks, n (%)	51 (27)	52 (41)
3–4 weeks, n (%)	33 (17)	17 (13)
> 1 month, n (%)	18 (9)	7 (6)
**Laboratory findings**		
Pleocytosis in CSF, median (range)^b^	143 (5–1280)	163 (5–634)
Anti-*Borrelia* antibodies in CSF, n (%)^c^	149 (77)	80 (63)
**LNB Diagnosis**		
Definite LNB, n (%)	149 (77)	79 (62)
Possible LNB, n (%)	45 (23)	48 (38)
**Persistent signs and symptoms at the 2-month follow-up**^a^		
Facial nerve palsy, n (%)	26 (13)	25 (20)
Headache, n (%)	9 (10)	5 (4)
Fatigue, n (%)	5 (3)	7 (6)
Abducens nerve palsy, n (%)	1 (1)	0 (0)
Affected balance, n (%)	1 (1)	0 (0)
Hemiparesis, n (%)	1 (1)	0 (0)
Shoulder pain, n (%)	1 (1)	0 (0)
Joint pain, n (%)	0 (0)	1 (1)
Neck pain, n (%)	0 (0)	1 (1)
Radiant pain in extremity, n (%)	0 (0)	1 (1)
Hyperesthesia in extremity, n (%)	0 (0)	1 (1)
**Clinical outcome at the 2-month follow-up**		
Recovered, n (%)	155 (80)	94 (74)
Not recovered, n (%)	39 (20)	33 (26)

^a^ Some patients have reported several symptoms

^b^Pleocytosis: > 5 × 10^6^/L white cells with a mononuclear cell dominance

^c^ Intrathecal *Borrelia* specific antibody synthesis in CSF

*LNB* Lyme neuroborreliosis

*CSF* Cerebrospinal fluid

In the ceftriaxone group, 149 patients (77%) were classified as definite LNB and 45 patients (23%) as possible LNB. In the doxycycline group, 79 patients (62%) were classified as definite LNB and 48 patients (38%) as possible LNB (Table 2). There was a clear statistically significant difference (*p* = 0.006) between groups. Among patients in the ceftriaxone group, 155 out of 194 (80%), were defined as having a complete recovery and 39 patients (20%) were defined as having an incomplete recovery at the 2-month follow-up (Fig. 1). In the doxycycline group, 94 patients (74%) were completely recovered and 33 patients (26%) were defined as having incomplete recovery (Fig. 1). The most commonly reported persistent signs or symptoms at the 2-month follow-up, in both groups, were facial nerve palsy, headache and fatigue. Clinical and laboratory characteristics on admission and at follow-up are shown in Table 2.

There was no significant difference in clinical outcome at the 2-month follow-up (recovery/non-recovery) when comparing children treated for LNB with either intravenous ceftriaxone or oral doxycycline (*p* = 0,217). When all relevant clinical and laboratory data from participants in the study (*n* = 317) were included and analyzed in a logistic multivariate regression analysis, there was still no significant association between antibiotic treatment and clinical outcome (OR 1.05 with 95%; CI 0.51–2.17) (Table 3). Additionally, there were no significant associations between age, gender, known tick bite, erythema migrans, headache, fatigue or pleocytosis on admission and clinical outcome at the 2-month follow-up (Table 3). However, facial nerve palsy (OR 2.72 with 95% CI 1.29–5.79) and fever (OR 2.36 with 95% CI 1.24–4.50) were associated with poorer clinical outcome (non-recovery), whereas occurrence of anti-*Borrelia*-antibodies in CSF (i.e. patients classified as definite LNB) was associated to better clinical outcome (recovery) (OR 0.41 with 95% CI 0.21–0.80) (Table 3).

**Table 3:** Results of the logistic multivariate regression analysis for relevant clinical and laboratory data in association to clinical outcome at the 2-month follow-up (recovery/non-recovery)

	***p***-value	Odds ratio (OR)	95% Confidence Interval (CI)
Age	0.547	1.03	(0.94–1.13)
Gender, male	0.490	1.22	(0.69–2.18)
Known tick bite	0.256	0.72	(0.41–1.27)
Erythema migrans	0.672	0.87	(0.45–1.68)
Facial nerve palsy	**0.009**	**2.73**	(1.29–5.78)
Headache	0.872	1.06	(0.54–2.08)
Fatigue	0.685	0.87	(0.44–1.70)
Fever	**0.009**	**2.36**	(1.24–4.50)
Pleocytosis ^a^	0.951	1.00	(1.00–1.00)
Anti-*Borrelia*-antibodies ^b^	**0.008**	**0.41**	(0.21–0.80)
Antibiotic treatment, ceftriaxone	0.887	1.05	(0.51–2.17)

^a^ Pleocytosis: > 5 × 10^6^/L white cells in CSF with a mononuclear cell dominance

^b^ Intrathecal *Borrelia* specific antibody synthesis in CSF

*CSF* Cerebrospinal fluid

## Discussion

In this large retrospective study on pediatric LNB patients, we have shown that there was no difference between children, independent of age, who had received intravenous ceftriaxone and those who had received oral doxycycline when comparing clinical outcome (recovery/non-recovery). Our results are in line with previous studies [19], supporting the hypothesis that oral doxycycline is as effective as intravenous ceftriaxone for treatment of LNB. However, the efficacy and safety of the two different treatment strategies could not fully be evaluated in our study, since it was not a randomized comparative study, and unknown confounding factors may have influenced our results.

One strength of our retrospective study was that results are based on data from a relatively larger patient sample (*n* = 321) including three previous prospective cohorts. Patients are well characterized and could probably be considered as representative of Swedish pediatric LNB patients. All participating children were clinically followed-up at 2 months, in all three cohorts. The follow-up visits were congruent and well executed by physicians at each pediatric department, including a clinical examination and a pre-defined structured questionnaire for self/parent-reported persistent symptoms. Patients were defined as being recovered/not-recovered based on findings from the examination and answers from the questionnaires. Unfortunately, for the assessment of clinical outcome, no clinical composite score nor validated questionnaire was used at the follow-up visits, which is a weakness of the study. However, we believe that the overall clinical evaluation of each patient, by pediatricians at the 2-month follow-up visit, was correct and sufficient to determine if the patient was recovered/not-recovered.

An additional limitation of the study is that we did not have precise data on the duration of antibiotic treatment, since children could have received a course of antibiotic treatment varying from 10 to14 days. Therefore, analysis of the association between treatment duration and clinical outcome was not feasible.

The duration of antibiotic treatment in children with early LNB has been under debate [23] and treatment for 10–30 days has been suggested [19, 24]. Recently published evidence-based guidelines from Germany have determined a recommendation of 14 days of doxycycline, intravenous ceftriaxone, cefotaxime or Penicillin G [23].

Furthermore, the safety of doxycycline concerning dental staining, given to children under 8 years of age, has been under debate [12–14] and recent guidelines have come to different recommendations. German guidelines from 2020 have kept the recommendation of doxycycline to children 9 years of age and up [23], while guidelines from the American Academy of Neurology have stated that oral doxycycline may be considered over intravenous treatment in children of all ages who can tolerate oral antibiotics [25].

We have chosen to include patients with both definite LNB and possible LNB in our large retrospective study. Admittedly, some of patients in the possible LNB group might have had some other diagnosis, which could possibly have influenced follow-up results. However, possible LNB patients in our study are well characterized, had no signs or laboratory findings of other diseases and responded well to antibiotic treatment. Furthermore, no difference in duration of symptoms on admission were found between definite LNB and possible LNB groups.

The age of the children and the choice of antibiotic treatment was not always congruent with Swedish guidelines in our study. Thirtyeight (*n* = 38) children had received intravenous ceftriaxone even though they were ≥ 8 years of age.These patients could possibly have had a more severe LNB on admission than other children. However, with the regression analysis, including age, symptoms on admission and antibiotic treatment, this should not have influenced our results on clinical outcome. In addition, when excluding patients who received both ceftriaxone and doxycycline (*n* = 4), we found no difference in results. Furthermore, we saw a higher percentage of treatment with intravenous ceftriaxone in the definite LNB group, as compared to the possible LNB group (*p* = 0.006), which may reflect the fact that pediatricians possibly favor ceftriaxone as treatment for LNB.

The most common persistent symptom at the 2-month follow-up in our study was facial nerve palsy (51 out of 211, 24%). Results were similar, with no statistically significant difference, in both diagnostic groups (definite LNB and possible LNB) and in both treatment groups (intravenous ceftriaxone and oral doxycycline). Results are in line with previous studies [10, 26]. The wast majority of patients with acute facial nerve palsy in this study came to hospital within 1–2 days and started antibiotic treatment the same day, directly after diagnostic evaluation with serum samples and lumbar puncture. Duration of symptoms before start of treatment should not have influences recovery negatively. In addition, any facial nerve impairment may further spontaneously improve until one year after the acute episode. Unfortunately, the follow-up period in our study was restricted to 2 months.

Facial nerve palsy on admission was also one of the major clinical manifestations associated with a higher risk of non-recovery in our logistic multivariate regression analysis. This result is not surprising, and in line with earlier studies, where the validated House-Brackmann grading scale has also been used to evaluate clinical outcome [26]. However, the manifestation of fever on admission and its association with poorer clinical outcome was more surprising. This association could possibly be understood, as the fever itself being a sign of strong immunological activity in CSF in LNB, and the inflammation could negatively influence both the ability of clearing symptoms and clinical outcome [27]. Anti-*Borrelia* antibodies in CSF were, contrary to facial nerve palsy and fever, associated with a better clinical outcome, possibly because of a faster and more determined decision for the start of treatment by the pediatricians in charge, which could have been beneficial for clinical recovery [10]. Admittedly, the discussion about these associations is somewhat speculative and in some measure difficult to explain from a pathophysiological point of view, and should be interpreted with caution.

## Conclusion

In this large retrospective study, no difference in clinical outcome (recovery/non-recovery) was found, independent of age, when comparing children who received intravenous ceftriaxone with children who received oral doxycycline, supporting an equal effectiveness for treatment of pediatric LNB patients. However, future randomized comparative treatment studies with non-inferiority design are warranted for evaluation of efficacy and safety of antibiotic treatment in pediatric LNB patients.

## Data Availability

The dataset used and/or analyzed during the current study are available from the corresponding author on reasonable request.

## Abbreviations

CI: Confidence interval
CNS: Central nervous system
CSF: Cerebrospinal fluid
LB: Lyme borreliosis
LNB: Lyme neuroborreliosis
